# Machine learning coarse grained models for water

**DOI:** 10.1038/s41467-018-08222-6

**Published:** 2019-01-22

**Authors:** Henry Chan, Mathew J. Cherukara, Badri Narayanan, Troy D. Loeffler, Chris Benmore, Stephen K. Gray, Subramanian K. R. S. Sankaranarayanan

**Affiliations:** 1 Center for Nanoscale Materials, Argonne National Laboratory, Argonne, IL 60439 USA; 2 X-ray Science Division, Argonne National Laboratory, Argonne, IL 60439 USA; 3 Department of Mechanical Engineering, University of Louisville, Louisville, KY 40292 USA; 4 Consortium for Advanced Science and Engineering, University of Chicago, Chicago, IL 60637 USA

## Abstract

An accurate and computationally efficient molecular level description of mesoscopic behavior of ice-water systems remains a major challenge. Here, we introduce a set of machine-learned coarse-grained (CG) models (ML-BOP, ML-BOP_dih_, and ML-mW) that accurately describe the structure and thermodynamic anomalies of both water and ice at mesoscopic scales, all at two orders of magnitude cheaper computational cost than existing atomistic models. In a significant departure from conventional force-field fitting, we use a multilevel evolutionary strategy that trains CG models against not just energetics from first-principles and experiments but also temperature-dependent properties inferred from on-the-fly molecular dynamics (~ 10’s of milliseconds of overall trajectories). Our ML BOP models predict both the correct experimental melting point of ice and the temperature of maximum density of liquid water that remained elusive to-date. Our ML workflow navigates efficiently through the high-dimensional parameter space to even improve upon existing high-quality CG models (e.g. mW model).

## Abstract (web summary)

A computationally efficient description of ice-water systems at the mesoscopic scale is challenging due to system size and timescale limitations. Here the authors develop a machine-learned coarse-grained water model to elucidate the ice nucleation process much more efficiently than previous models.

## Introduction

Ice nucleation and grain growth are ubiquitous phenomena. Ice nuclei, when formed, are nanoscopic^1^—critical sizes start from tens of molecules—and subsequently consolidate into larger grains at the mesoscopic scale^2^. A molecular level picture of phase transformations in water, especially at mesoscopic scales, is most desirable but remains inaccessible to fully atomistic simulations^3^. The underlying phase transitions and dynamical processes in supercooled mesoscale systems are often inaccessible due to system size and timescale limitations, which are further compounded by their sluggish kinetics. While exascale computers may cope with such mesoscopic length scales, time scale challenges will remain (Supplementary Figure 1). It is important to have a water model that accurately captures the melting point, liquid and solid densities, as well as other thermodynamic and transport properties at modest computational cost. Numerous atomistic^4–6^ and coarse-grained^7^ (CG) water models exist. They differ in terms of predictive power and computational cost/efficiency. The best performing non-polarizable atomistic model is TIP4P/2005^5^. However, it under-predicts the melting point by 20 K and is too computationally expensive for large-scale molecular dynamics (MD) studies involving multi-million molecule ice-water systems. Polarizable models such as MB-pol^8^ and AMOEBA^9^ have comparable accuracy and can treat charged species, but are computationally expensive (Tables 1 and 2). CG models are computationally efficient, but often less accurate. The monoatomic water (mW) model^10^ remains the best performing CG model^11^, predicting the correct melting point and several thermodynamic properties, but does not quantitatively capture the density anomaly and over predicts density of ice (Supplementary Figure 2).

**Table 1:** Performance of ML models compared to other popular water models

	ML-BOP	ML-BOP /dih	ML-mW	mW	TIP4P /2005	MB-pol	iAMOEBA	SPC/E	TIP3P
Neighbors_3.3 Å, 298 K_	9	9	9	10	9	9	9	9	10
res_RDF, 298 K_	3	4	8	8	7	9	7	6	0
res_ADF, 298 K_	7	7	7	7	6	7	7	6	6
ln D_298 K_	5	5	0	0	8	9	8	8	0
ρ_298K, 1atm_^a^	10	10	10	10	9	8	10	9	7
ρ_max_^a^	10	10	10	9	10	7	10	8	2
TMD^a^	10	10	10	6	10	7	10	5	0
ΔH_vap_	8	8	9	9	4	8	8	6	10
T_m_	10	10	8	10	7	9	7	1	0
TMD - T_m_	10	10	7	4	6	8	7	6	4
ΔH_melt_^a^	7	7	9	8	6	–	7	0	0
ΔS_melt_	7	7	8	7	7	–	7	3	0
ρ_liq at Tm_^a^	10	10	10	10	9	7	10	8	6
ρ_Ih at Tm_^a^	7	7	8	0	9	9	7	3	3
ΔV_melt_	7	7	7	0	8	8	7	4	6
(dp/dT)_melt_	9	10	9	0	10	–	9	8	0
**Average score**	**8.1**	**8.2**	**8.1**	**6.1**	**7.8**	**8.1**	**8.2**	**5.6**	**3.4**

Comparison of the performance of ML models with other popular polarizable^8,19^ and non-polarizable models^26^. The numerical scores and tolerance are assigned based on an established system by Vega^59^. A list of ice and liquid water properties relevant to the capability of ML-BOP models are selected for comparison

^a^Properties that are included in the training of ML models

**Table 2:** Comparison of the computational cost for water models

Model	Cost in core-sec for 10 ps
mW	3.6
ML-BOP	3.8
ML-BOP_dih_	5.9
ML-mW	2.5
TIP4P/2005	400.0
MB-pol	3213650.0
AMOEBA	1550.0
TIP4P/Ew	410.4
SPC/E	185.6
TIP3P	184.4

The benchmark system is liquid water (256 molecules) at 298 K

**Fig. 1:**
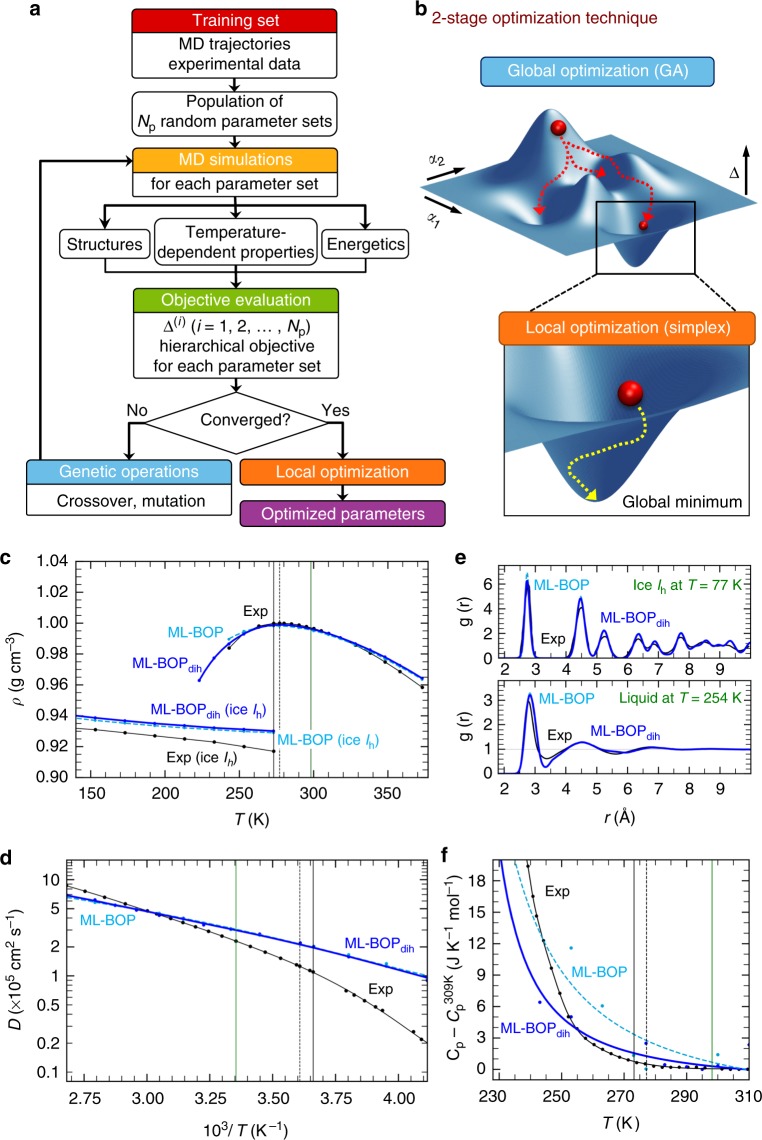
Machine learning protocol to train water potentials and comparison with experiments. **a** Workflow depicting force field parameterization. One novelty is a direct fitting to dynamically-inferred properties through long time scale MD simulations. *N*_p_ refers to population size and Δ^(i)^ refers to errors computed for the *i*^th^ parameter set in the *N*_p_ population using hierarchical objective. **b** Diagrams illustrating the 2-stage technique for locating the global minimum of the objective landscape. (The actual optimization involves up to 17 parameters but here we indicate just two generic parameters, α_1_ and α_2_.) Table 3 has the optimized ML-BOP and ML-BOP_dih_ parameters. In (**c**–**f**) the experimental (Exp) melting point (*T* = 273 K), maximum density temperature (*T* = 277 K), and room temperature (*T* = 298 K) are vertical solid black, dotted black, and solid green lines, respectively. **c** ML-BOP models accurately reproduce the density anomaly of water within 1.4% as shown by comparison with experimental densities^55^ of ice and liquid water at pressure 1 bar. Melting point of ML-BOP models is 273 ± 1 K. **d** ML-BOP models predict the experimental diffusion coefficients of water^20,56^ over a wide temperature range. **e** ML-BOP models reproduce the experimental radial distribution functions of ice at *T* = 77K^57^ and liquid water at *T* = 254 K^23^. **f** ML-BOP models capture the experimental heat capacity of water^58^ relative to the value at *T* = 309 K

A correct description of water’s complex properties with a potential model, especially in CG form, is challenging. Here, we introduce a machine-learning (ML) workflow (Fig. 1) that can be used to train models that accurately describe the behavior of ice and liquid water at mesoscopic scales. We develop a set of bond-order CG models (ML-BOP and ML-BOP_dih_) that are up to two orders of magnitude cheaper (Table 2, Supplementary Figure 3) than the most accurate non-polarizable atomistic models (TIP4P models and TIP5P) of comparable accuracy. As with the mW model^10^, our models treat each water molecule as one bead; the interactions between the beads are treated using a bond-order potential (BOP) both with and without explicit four-body term, i.e., on-the-fly dihedrals to describe tetrahedral solids. We use a multi-level hierarchical global optimization strategy to navigate the high-dimensional parameter space and train the ML models. We introduce ML models that adequately describe the thermodynamic and dynamical properties of water. Moreover, we also demonstrate that our ML strategy can be used to re-optimize existing high-quality water models, such as mW, and improve their overall performance.

## Results

### Machine learning workflow for training CG water models

The ML workflow to train CG models involves three main aspects: Model selection, Training data generation, and Multi-level hierarchical objective optimization to parameterize models against target training data. The various stages involved in the ML workflow are discussed below.

### CG model of water

Water molecules are modeled using a 1:1 CG model. The mapping of atomistic water molecules into CG water beads is done via the removal of hydrogen atoms, such that the CG beads are positioned at the positions of the oxygen atoms. This representation of water molecules as monoatomic beads and the use of the ML models can lead to a much more significant speed-up in MD simulations than the naive factor of three from the spatially reduced number of atoms. This is a result of larger simulation time steps being possible due to the absence of fast O–H vibrations, a significantly reduced number of pairwise interactions due to the reduced number of atoms, and the very simple CG potential form. A 1:1 CG model of water achieves both simplicity and computational efficiency.

### Machine learned bond-order potential for CG water

The ML-BOP model is based on the Tersoff-Brenner formalism^12^ (Pauling bond-order concept), which is used here to describe the short-range directional interactions between CG water beads. The pair potential function *V*_pair_ is given by

1 $$V_{{\mathrm{pair}}} = {\mathrm{f}}_{\mathrm{C}}\left( {r_{ij}} \right)\left[ {{\mathrm{f}}_{\mathrm{R}}\left( {r_{ij}} \right) + {\mathrm{b}}_{ij}{\mathrm{f}}_{\mathrm{A}}\left( {r_{ij}} \right)} \right]$$

where f_C_(*r*_*ij*_), f_R_(*r*_*ij*_), and f_A_(*r*_*ij*_) are the cutoff, repulsive, and attractive pair interactions, respectively, between bead *i* and *j* separated by a distance *r*_*ij*_, and b_*ij*_ is a bond-order parameter which modifies the pair interaction strength between bead *i* and *j* depending on their local chemical environment.

The cutoff function limits the range of interaction mainly to improve computational efficiency. The function is given by

2 $${\mathrm{f}}_{\mathrm{C}}\left( r \right) = \left\{ {\begin{array}{cc} 1, & r \,< \,R - D \\ \frac{1}{2} - \frac{1}{2}\sin \left( {\frac{{{\mathrm{\pi }}(r - R)}}{{2D}}} \right), & R - D \,< \,r \,< \,R + D \\ 0, & r \,> \, R + D \end{array}} \right.$$

where *R* and *D* are free parameters that are chosen to include only the first nearest neighbors, such that their pair interactions are smoothly reduced starting from the distance *R*−*D* and are completely turned off beyond the distance *R*+*D*.

The repulsive and attractive pair interactions between CG water beads are modeled using exponential decay functions given by

3 $${\mathrm{f}}_{\mathrm{R}}\left( r \right) = A{\mathrm{e}}^{ - \lambda _1r}$$

4 $${\mathrm{f}}_{\mathrm{A}}\left( r \right) = - B{\mathrm{e}}^{ - \lambda _2r}$$

where *A*, *B*, *λ*_1_, and *λ*_2_ are free parameters that control the overall strength and length scale of the repulsive and attractive potentials. Furthermore, the strength of f_A_(*r*) between beads *i* and *j* is scaled by a bond-order term b_*ij*_ which is given by

5 $${\mathrm{b}}_{ij} = (1 + \beta ^n{\mathrm{\xi }}_{ij}^n)^{ - \frac{1}{{2n}}}$$

6 $${\mathrm{\xi }}_{ij} = \mathop {\sum}\nolimits_{k \ne i,j} {{\mathrm{f}}_{\mathrm{C}}\left( {r_{ik}} \right){\mathrm{g}}\left( {\theta _{ijk}} \right)}$$

7 $${\mathrm{g}}\left( \theta \right) = 1 + \frac{{c^2}}{{d^2}} - \frac{{c^2}}{{[d^2 + (\cos \theta - \cos \theta _0)^2]}}$$

where *β*, *n*, *c*, *d*, and cos*θ*_0_ are free parameters. ξ_*ij*_ defines the effective coordination of bead *i*, taking into account the relative distances r_*ik*_ and interatomic angles *θ*_*ijk*_ of its neighboring beads. The three-body angular dependence is described by the function g(*θ*), which has minima defined by cos*θ*_0_ and the strength and sharpness of its effect controlled by *c* and *d*.

### Machine learned bond-order potential with on-the-fly dihedrals

Typical potentials (e.g., Stillinger-Weber or bond-order based such as Tersoff as described above) are based on first nearest neighbor interactions and hence the functional forms do not explicitly distinguish (energetically) between cubic and hexagonal ice structures. To address this limitation, we extend the Tersoff bond-order potentials to include on-the-fly dihedral calculations similar to that implemented in AIREBO type models^13^. The dihedral potential function is described by

8 $$V_{{\mathrm{dihedral}}}(\varphi ) = k_{{\mathrm{dih}}}\left[ {{\mathrm{sin}}^{3p}\left( {\frac{\varphi }{2}} \right) - {\mathrm{cos}}^p\varphi } \right]$$

where *k*_dih_ is the minimum well-depth, *p* controls the steepness of the well, and *φ* is the dihedral angle. In contrast to the dihedral potential functions typically used in rigid-bond models, the well-depth (and number of minima) of this potential changes depending on the number and local coordination of water beads. To improve computational efficiency and to handle any discontinuities due to this reactive characteristic, the Tersoff cutoff function, f_C_(*r*), is applied to every pair of water beads constituting a dihedral angle and an angular cutoff function, f_D_(*θ*), is applied to every triplet of those water beads.

9 $${\mathrm{f}}_{\mathrm{D}}\left( \theta \right) = \left\{ {\begin{array}{cc} 1, & \cos \theta _{2{\mathrm{a}}} \le \cos \theta \le \cos \theta _{1{\mathrm{b}}} \\ {\mathrm{t}}_1^2\left( {3 - 2{\mathrm{t}}_1} \right), & \cos \theta _{1{\mathrm{b}}} < \cos \theta < \cos \theta _{1{\mathrm{a}}} \\ 1 - {\mathrm{t}}_2^2\left( {3 - 2{\mathrm{t}}_2} \right), & \cos \theta _{2{\mathrm{b}}} < \cos \theta < \cos \theta _{2{\mathrm{a}}} \\ 0, & \cos \theta _{1{\mathrm{a}}} \le \cos \theta \le \cos \theta _{2{\mathrm{b}}} \end{array}} \right.$$

10 $${\mathrm{t}}_1 = \frac{{\cos \theta - \cos \theta _{1{\mathrm{a}}}}}{{\cos \theta _{1{\mathrm{b}}} - \cos \theta _{1{\mathrm{a}}}}},{\mathrm{t}}_2 = \frac{{\cos \theta - \cos \theta _{2{\mathrm{a}}}}}{{\cos \theta _{2{\mathrm{b}}} - \cos \theta _{2{\mathrm{a}}}}}$$

where cos*θ*_1a_ and cos*θ*_2b_ define the lower and upper bounds of the angular cutoff analogous to *R*+*D* in f_C_(*r*), cos*θ*_1b_ and cos*θ*_2a_ define the switching angle for the lower and upper bound angular cutoffs analogous to *R***−***D* in f_C_(*r*).

### Model parameterization

The parameterization of ML-BOP for water requires simultaneous optimization of 11 free parameters (*R*, *D*, *A*, *B*, *λ*_1_, *λ*_2_, *β*, *n*, *c*, *d*, cos*θ*_0_). Likewise, the parameterization of ML-BOP_dih_ for water requires optimization of 17 free parameters (*R*, *D*, *A*, *B*, *λ*_1_, *λ*_2_, *β*, *n*, *c*, *d*, cos*θ*_0_, cos*θ*_1a_, cos*θ*_1b_, cos*θ*_2a_, cos*θ*_2b_, *k*, *p*), which makes independent fitting of the parameters infeasible. Most of these parameters do not correspond to physical properties of the system, so they cannot be chosen based on intuition. In this work, we employ global and local optimization techniques and state-of-the-art machine learning principles to search for an optimized parameter set for water as described below.

### Multi-level hierarchical objective machine learning workflow

Our machine learning workflow to train the CG models is illustrated in Fig. 1. In our training scheme (Fig. 1a), we introduce a multilevel evolutionary strategy (hierarchical objective genetic algorithm—HOGA) to train the ML models against an extensive training data set of energies and structural properties of ice and liquid water derived from the best available atomistic model (TIP4P/2005), supplemented by experimental data. The training data in the case of ML-BOP_dih_ also includes first principles energetic differences reported for cubic and hexagonal ice phases^14^. This elaborate training data set ensures an adequate representation of the diverse configurational space of ice and liquid water while amply sampling the energy landscape. We use HOGA to perform a global search followed by local optimization to find the optimized model parameters (Fig. 1b). This circumvents problems encountered with the local minimizers often used in force field fitting that rely on good starting guesses. An important new aspect of our scheme is that the iterations involve not just static evaluations of potential properties but also temperature-dependent properties sampled dynamically from several MD trajectories during the evolutionary process (10’s of milliseconds of overall MD trajectories). HOGA aids in an accelerated evolutionary search by efficiently sampling the parameter landscape within a given GA generation, and overcoming the limitation of assigning arbitrary weights within a single objective thereby ensuring that all the properties (static or dynamic) are equally well described.

### Training data set

The machine learning workflow begins with the preparation of an extensive data set, which is necessary for a supervised training method. We build the training set from atomistic MD trajectories of 1600 TIP4P/2005 water molecules, simulated at pressure *P* = 1 bar over a wide range of temperatures using the LAMMPS simulator^15^ with a 13 Å interaction cutoff, the particle-particle particle-mesh method for long-range electrostatic interactions, and a 1 fs time step. The training set consists of various ice and liquid water configurations, which includes hexagonal ice at 123 K < *T* < 273 K, supercooled liquid water at 253 K < *T* < 273 K, normal condensed phase liquid water at 273 K < *T* < 373 K, and ice-water interfaces. Unlike most typical force field fitting procedures, we fit to the structure and energetics of TIP4P/2005 water configurations in the training set but also go beyond that by using the TIP4P/2005 training set as good starting configurations for running MD simulations with ML models during the fitting process. Properties including dynamical properties can be sampled from these simulations and be used to fit directly to experimental values of thermodynamic properties. Note that the main limitation of the TIP4P/2005 model is its inability to get the correct melting point (*T*_m_) and the relative difference between temperature of maximum density (TMD) and *T*_m_. In such cases, we use known experimental values as targets.

All MD simulations performed during our force field fitting workflow are run in an isobaric-isothermal ensemble at pressure *P* = 1 bar and different target temperatures using the LAMMPS simulator^15^. The equilibration time of these simulations varies from 150 ps to 4 ns depending on the configuration and temperature (e.g., shortest for ice and longest for supercooled water). Note that the training set contains configurations of ice and liquid water over a wide range of temperatures, static properties as well as time-averaged properties such as ΔH_m_ and ρ_i_ sampled from MD simulations.

### Hierarchical objective genetic algorithm (HOGA)

The quality of a proposed parameter set is evaluated based on a hierarchical objective function (see pseudo code in Supplementary Note 1). In the HOGA evolutionary scheme, we truncate the evaluation of a parameter set which leads to large errors in hierarchical property classes and assign it a penalty depending on which class it fails at. The selection of hierarchical classes is at the discretion of the user. In this case, the hierarchy of the property classes is as listed in Supplementary Table 2. Note that a higher preference is given to the temperature-dependent densities of ice, water and the melting point to ensure that the models reproduce the density anomaly and the relative locations of melting point and TMD. The hierarchical approach aids in an accelerated evolutionary search by efficiently sampling the parameter landscape within a given generation, and overcoming the limitation of assigning arbitrary weights within a single objective thereby ensuring that all the properties (static or dynamic) are equally well described.

Given the objective function definition as described above, we proceed to apply a two-stage optimization technique to search for a suitable parameter set for water in the multi-dimensional parameter space. The goal is to locate the global minimum in the objective value landscape. We strategically start with a broad survey of the landscape using global optimization methods followed by a deeper refinement search using local optimization methods. In principle, any combination of global and local optimization methods should work for such a workflow. Here, we choose to use the genetic algorithm^16^ (GA) for global optimization and the Nelder-Mead simplex algorithm^17^ for local optimization.

Using HOGA, the global optimization process begins with the initialization of a population of *N*_p_ random parameter sets. The objective value Δ^(i)^ for each of these parameter sets is evaluated and their convergence is checked. If the convergence criteria are not met, then a new list of *N*_p_ parameter sets is derived using genetic operations (selection, cross-over, mutation, etc.) from the m old parameter sets having the lowest objective values. The selection operation creates a list of best parameter sets based on their objective values, which mimics the principle of “survival of the fittest” in evolution. The crossover operation intermixes these parameter sets to generate new potential good candidates, analogous to how good traits are passed from biological parents to their offspring. The mutation operation introduces sufficient diversity into the population to avoid pre-mature convergence of the GA run, which also provides the population the opportunity to improve beyond those possible via inheriting traits from parent structures (crossover). In this work, we used tournament selection without replacement as the selection operation, the simulated binary method as the crossover operation with an operation probability of 0.9, and a polynomial of order 20 for the mutation operation with an operation probability of 0.1. The objective value is evaluated for the new parameter sets followed by convergence test. This routine is iteratively performed until convergence.

To effectively survey the objective landscape, we typically perform at least 20 GA runs simultaneously (up to a total of 100 runs), where each GA run has a population size of 200 and run for about 100 generations. The global optimization stage typically returns a list of close-to-optimal parameter sets which we further refine using local optimization techniques. In this work, we use the Nelder-Mead simplex algorithm^17^ for local optimization, and the final parameter set is chosen based on the performance in validation tests. The best parameter sets for ML-BOP and ML-BOP_dih_ optimized through HOGA are provided in Table 3.

**Table 3:** Force field parameters of ML models optimized using our developed workflow

*ML-BOP*					
*m* ^a^	Gamma ^a^	*λ*_3_ (Å^−1^) ^a^			
1.0	1.0	0.0			
*C*	*d*	cos*θ*_0_	*n*	*β*	
77638.534354	16.148387	−0.471029	0.770018	1e-06	
*λ*_2_ (Å^−1^)	*B* (eV)	*R* (Å)	*D* (Å)	*λ*_1_ (Å^−1^)	*A* (eV)
2.199640	473.621419	3.282761	0.270511	2.750522	1684.301476
*ML-BOP*_*dih*_					

*m* ^a^	Gamma ^a^	*λ*_3_ (Å^−1^) ^a^			
1.0	1.0	0.0			
*C*	*d*	cos*θ*_0_	*n*	*β*	
77638.534354	16.148387	−0.471029	0.770018	1e-06	
*λ*_2_ (Å^−1^)	*B* (eV)	*R* (Å)	*D* (Å)	*λ*_1_ (Å^−1^)	*A* (eV)
2.199640	473.621419	3.282761	0.270511	2.750522	1684.301476
cos*θ*_1a_	cos*θ*_1b_	cos*θ*_2a_	cos*θ*_2b_	*k*_dih_ (eV)	*p*
0.156434	0.017452	−0.390731	−0.5	0.2e-3	8
*ML-mW * ^b^					

*ϵ* (eV)	*σ* (Å)	*a*	*λ*	*γ*	cos*θ*_0_
0.297284	1.884015	2.124872	24.673877	1.207943	−0.279667
*A*	*B*	*p*	*q*	tol ^a^	
7.111598	1.991526	4.011214	0.0	0.0	

^a^Parameters that are not optimized in our ML workflow

^b^See Supplementary Equation 1–3 for the functional form (Stillinger-Weber, same as mW^10^)

### Model validation and performance of machine learned CG water models

Figure 1c, d compares structural and dynamics-inferred properties with experimental data. Our ML-BOP models successfully capture the best-known thermodynamic anomaly, the existence of a density maximum at 277 K (Fig. 1c); they correctly describe the freezing/melting transition at 273 ± 1 K, and densities of ice (140 K–273 K) and water (243 K–373 K) within 1.4% of experiments. Capturing the correct value of the TMD relative to the melting point has remained a challenge for all water models^10,18,19^. TIP4P/2005 is the best atomistic model to depict TMD but underestimates the melting point by 20 K. Regarding transport properties (Fig. 1d), the room temperature diffusivity, ML-BOP models is ~3 × 10^−5^ cm^2^ s^−1^ in close agreement with experiment^20^ (2.3 × 10^−5^ cm^2^ s^−1^). Both ML models slightly overestimate diffusivities in the supercooled range but outperform other existing water models (Supplementary Figure 2b).

Figure 1e compares the O–O radial distribution function (RDF) for ice I_h_ at 77 K and (supercooled) liquid water at 254 K derived from experiments. The location and intensities of the peaks corresponding to first, second and third coordination shells are in good agreement. ML-BOP models, however, over-structure water, and underestimate the exchange of water molecules between first and second coordination shell^21,22^ (deeper minimum in the radial distribution function or RDF at ~3.4 Å). Our model is suitable for mesoscopic phenomena, such as ice nucleation and grain growth as well as applications involving polycrystalline ice, e.g., friction, mechanics of ice, melting of ice crystals, or pollutant effects on nucleation and ice grain growth (for example, see Supplementary Figure 4). The model captures the temperature and pressure dependent (Supplementary Figure 6) trends of these peaks. The ML-BOP calculated number of water neighbors in the first solvation shell, integrated out to the predicted temperature independent isosbestic point (*r* = 3.25 Å), is 4.7 in accordance with the experimental range of 4.3–4.7^23,24^. Also, the angular distribution function at 298 K agrees well with TIP4P/2005 (Supplementary Figure 5b). The ML-BOP heat capacities for liquid water, with respect to their values at 309 K, reproduce the thermodynamic anomaly indicated by the sharp increase in C_p_ of supercooled water (Fig. 1f). We also introduce an ML-BOP_dih_ which represents a modification of ML-BOP model to include on-the-fly dihedrals. ML-BOP_dih_ performs on par with ML-BOP and additionally was trained using HOGA to capture the DFT predicted free energy difference (~1.4 meV/atom per water molecule) between ice polymorphs. The performances of both ML-BOP and ML-BOP_dih_ are detailed in Tables 4–6 and Supplementary Figure 2-6. Overall, the trained ML models perform better or on par with the best available water models in several of the properties listed, but at a fraction of the computational cost.

**Table 4:** Solid-liquid interfacial energies for hexagonal ice

	γ (mJ m^−2^)
Exp	29**–**33^a^
ML-BOP	26.3
ML-BOP_dih_	26.8
ML-mW	29.3
mW	35^b^
TIP4P/2005	29^b^
TIP4P/Ice	30^b^
TIP4P-Ew	37^c^
TIP5P	42^c^

^a^ref. ^60^

^b^ref. ^62^

^c^ref. ^61^

**Table 5:** Properties of ML models compared to experiments and other popular water models

	Exp	ML-BOP	ML-BOP/dih	ML-mW	mW	TIP4P/2005	MB-pol	iAMOEBA	SPC/E	TIP3P
Neighbors (3.3 Å cutoff)	4.51^a^	4.66	4.67	4.58	4.49	4.44	4.58	4.46	4.41	4.55
D_298 K_ (×10^−5^ cm^2^ s^−1^)	2.3^b^	3.0	3.0	4.7	6.4	2.1	2.2	2.5	2.5	5.2
ρ_298K,1atm_ (kg m^−3^)^b^	997.0	995.6	996.5	997.0	997.3	993	1007	997	994	982
ρ_max_ (kg m^−3^)^c^	999.9	998.3	999.0	998.5	1003.8	1001	1014	999.9	1012	1038
TMD (K)^c^	277	276	278	279	251	278	258	277	241	182
ΔH_vap_ (kcal mol^−1^)	10.52	10.01	10.01	10.30	10.66	11.98	10.1	10.94	11.69	10.49
T_m_ (K)	273	273	273	289	273^d^	252	264	261	215	146
TMD—T_m_ (K)	4	3	5	−10	−24	26	−6	16	26	36
ΔH_melt_ (kcal mol^−1^)^c^	1.44	1.23	1.23	1.40	1.26	1.16	–	1.19	0.74	0.30
ΔS_melt_ (cal mol^−1^K^−1^)	5.27	4.52	4.52	4.84	4.60	4.6	–	4.56	3.44	2.06
ρ_liq_ at T_m_ (kg m^−3^)^c^	999.8	997.95	998.0	998.5	1001.0	993	1013	999	1011	1017
ρ_Ih_ at T_m_ (kg m^−3^)^c^	917	929	930	928	978	921	920	929	950	947
ΔV_melt_ (cm^3^ mol^−1^)	−1.61^e^	−1.35	−1.39	−1.38	−0.42	−1.42	−1.80	−1.36	−1.14	−1.31
(dp/dT)_melt_ (bar K^−1^)	−137^f^	−141	−136	−146	−463	−135	–	−141	−126	−66

^a^ref. ^23^

^b^ref. ^20^

^c^Properties that are included in the training of ML models

^d^ref. ^64^

^e^ref. ^63^

^f^ref. ^59^

Properties comparison from experiments^55^, popular polarizable^8, 19^ and non-polarizable models^26^

**Table 6:** Mean enthalpy and free-energy of various ice polytypes predicted by ML-BOP_dih_

Stacking	Mean enthalpy (eV/molecule)	Free energy, G (eV/molecule)	G - GIh (meV/molecule)
I_c_(ABCABC)	−0.39506	−0.50768487	0.959
I_h_ (ABABAB)	−0.39528	−0.50864357	0.000
ABABCB	−0.39526	−0.50809176	0.552
ABACBC	−0.39511	−0.50790687	0.737
ABCACB	−0.39509	−0.50793255	0.711
ABCBAB	−0.39526	−0.50808043	0.563
ABCBCB	−0.39523	−0.50810750	0.536

The mean enthalpy and free-energy (eV/molecule) are computed at 260 K. The free energy difference relative to the most stable hexagonal ice phase is also given

### HOGA to retrain existing best performing CG models

**Fig. 2:**
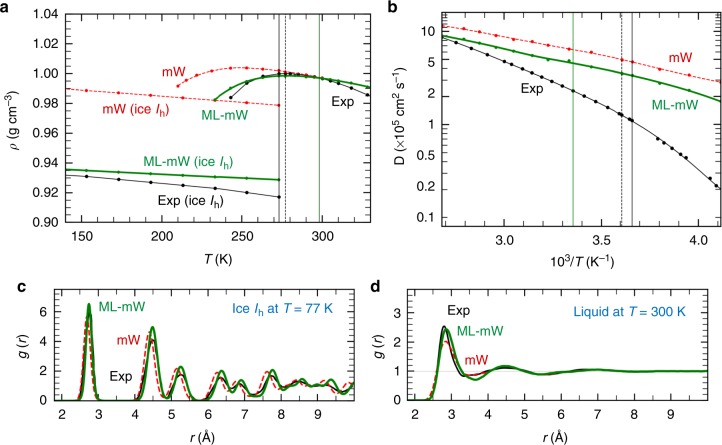
Comparisons of the predicted properties of ML-mW and the original mW with experiments. In (**a**–**b**) the experimental (Exp) melting point (*T* = 273 K), maximum density temperature (*T* = 277 K), and room temperature (*T* = 298 K) are vertical solid black, dotted black, and solid green lines, respectively. **a** Densities of ice and liquid water at pressure 1 bar. ML-mW melting point is 289 ± 1 K. **b** Diffusion coefficients of water over a wide temperature range. Radial distribution functions of **c** ice at *T* = 77 K and **d** liquid water at *T* = 300 K. Table 3 has the optimized ML-mW parameters

Our machine learning strategy is quite general and can be used to improve a variety of existing material models. To demonstrate this capability, we retrain the best available coarse grained model, for water, i.e. the mW model^10^, against our training data-set using the HOGA ML workflow. The new mW model trained using the machine learning workflow (termed ML-mW and given in Table 3) correctly captures the TMD, the density and structure of ice in the supercooled regime (140–270 K) as well as improves several thermodynamic and transport properties compared to original mW while retaining the structure (e.g., RDF) of liquid mW water. The limitation of the ML-mW is that the melting point is slightly over-predicted (~289 K) and, in contrast with the ML-BOP and ML-BOP_dih_ models, is unable to get the relative difference between *T*_m_ and TMD. Nevertheless, the overall predictions of ML-mW are better than the original mW in several properties (see Fig. 2 and Tables 1 and 5).

Briefly, the ML trained mW improves upon several of the properties compared to the original mW. For example, the diffusion coefficient at 300 K is 4.8 × 10^−5^ cm^2^ s^−1^, which is closer to the experimental value of 2.4 × 10^−5^ cm^2^ s^−1^. Likewise, the density of ice at the melting point improves from 0.978 to 0.930 g cm^−3^, which is closer to the experimental ice density (0.917 g cm^−3^). The volume change upon melting, the TMD as well as the enthalpy of melting show an improvement over the original mW model. Other properties such as (dp/dT)_melt_ also show a significant improvement as detailed in Table 5. Note that these improved predictions come by sacrificing the melting point; the ML-mW predicts the melting point to be 289 K which is 16 K higher than the original mW and experimental melting point. The HOGA algorithm is able to efficiently sample the high-dimensional parameter space and arrive at an optimal set of mW parameters with an improved overall score for the properties listed in Table 1.

### Origin of the improvement in the ML model performance

**Fig. 3:**
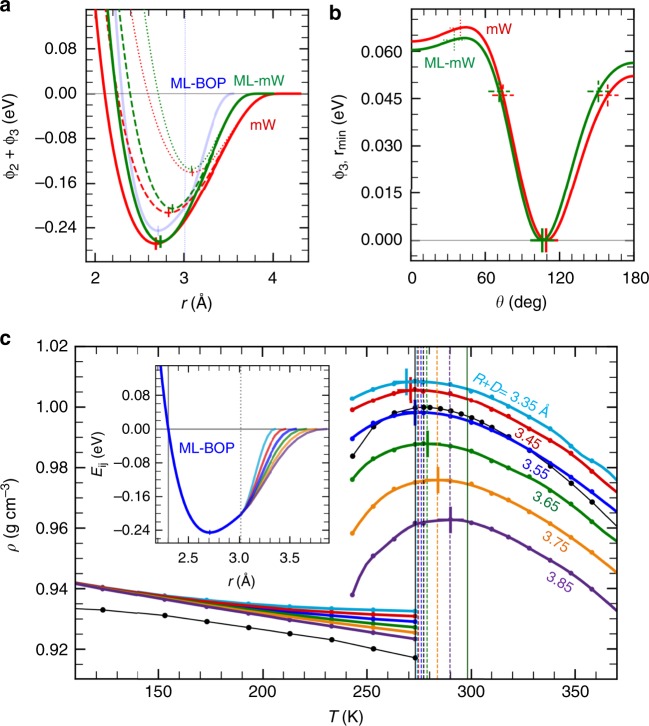
Origin of the performance improvements in the ML-mW and ML-BOP models. **a** Solid line style curves compare the 2-body only interaction energy of ML-mW, mW, and ML-BOP (ϕ_3_ = 0 for the mW models and b_*ij*_ = 1 for ML-BOP). The vertical dotted line marks the Tersoff cutoff switching distance (*R*−*D*) in ML-BOP. Dashed and dotted line style curves show the pair interaction energy of ML-mW and mW under the influence of a third atom, the addition of ϕ_3_. **b** 3-body energy term, ϕ_3_, evaluated at the minimum of the pair interaction energy curves, *r*_min_. Note that we first identify *r*_min_ for each interatomic angle *θ* and then compute the corresponding ϕ_3._ The cross marks indicate the *θ* at which the interaction energy curves in (**a**) are evaluated. **c** The explicit cutoff function, f_c_, in Tersoff provides a flexibility to independently modify the tail portion of the pair interaction energy curve (inset). This influences the relative separation between melting point (cross marks) and TMD (vertical dotted lines) in the temperature-dependent density plot

To elucidate the improvements of the ML-mW and ML-BOP models, we compare the pair-wise interaction energy curves of these two models with the original mW model (Fig. 3a). As seen in the energy curves representing only the 2-body interactions (solid line style), there are two notable differences as we go from mW to ML-mW to ML-BOP. There is a progressive steepening of the repulsive wall at *r* < 2.7 Å, and the interaction cutoffs become shorter (4.3 Å to 4.0 Å to 3.6 Å). A large increase in repulsive interaction can also be inferred from the ~3.3 times larger value of parameter *B* (coefficient of the repulsive term) in the functional form of ML-mW vs. mW (Supplementary Table 1). Furthermore, in contrast to a previous study that mW has the shortest optimal interaction cutoff necessary for capturing the anomalous properties of water^25^, HOGA is able to find a model with a shorter cutoff that improves the original model. The shorter cutoff of ML-mW also contributes to its improved efficiency over mW (Table 2). ML-mW has a 3° deviation (left shift of minimum in Fig. 3b) from the ideal tetrahedral angle of 109.47° (in mW), and has a larger 3-body energy penalty for interatomic angles *θ* >130° but a smaller penalty for *θ* < 70°. The dashed and dotted cross marks in Fig. 3b mark the interatomic angles (~37°, ~72°, ~155°) at which the dashed and dotted energy curves in (a) are evaluated. The overall effect of bond order in the two models appears similar. We note that there have been prior efforts by Molinero and co-workers at improving the parameterization of the mW model using relative entropy minimization^26^ (REM) as well as using uncertainty quantification^25^ (UQ). Both of these studies provide useful insights into the effect of model parameters on system properties. Note that while the search spaces in UQ were localized around the already optimized mW set, the parameter search in the REM procedure was global. In both the cases, the overall performance of those re-parameterized models were found to be poorer when compared to the original mW. In the present case, the performance improvements in ML-mW arise from a drastic deviation of potential parameters from the already optimized mW parameter set. This signifies the effectiveness of HOGA in navigating the high-dimensional parameter space and arriving at a set of optimal parameters that outperforms other optimization techniques such as REM and UQ.

Although both ML-mW and ML-BOP have quantitively improved the description of liquid density anomaly as well as the density of ice in mW, only ML-BOP (in fact out of all currently existing water models) is able to capture the correct ordering and the relative temperature difference between the melting point and TMD of water. A major difference is the use of explicit cutoff by the Tersoff functional form (ML-BOP models) as against the implicit cutoff functions employed by Stillinger-Weber form (mW models). An explicit cutoff function provides the flexibility to modify the tail portion of the pair interaction energy curve independent of the rest of features such as the repulsive wall, location and depth of minimum, etc. (see inset of Fig. 3c). As the ML-BOP cutoff (*R*+*D*) becomes smaller, while keeping the switching distance (*R*−*D*) fixed, the relative separation between the melting point (cross marks) and TMD (vertical dotted lines) reduces and their ordering eventually flips (Fig. 3c). We further note that the tail portion of the interaction energy curve also has a strong influence on other properties including the liquid densities, ice densities close to the melting point, enthalpy of melting, diffusion coefficients, etc., which exemplifies the challenge in simultaneously optimizing many properties (i.e., multi-objective) and the need of a ML workflow in place of the local optimization based fitting procedures and/or driven by human intuition.

### Simulations of ice nucleation in supercooled water

**Fig. 4:**
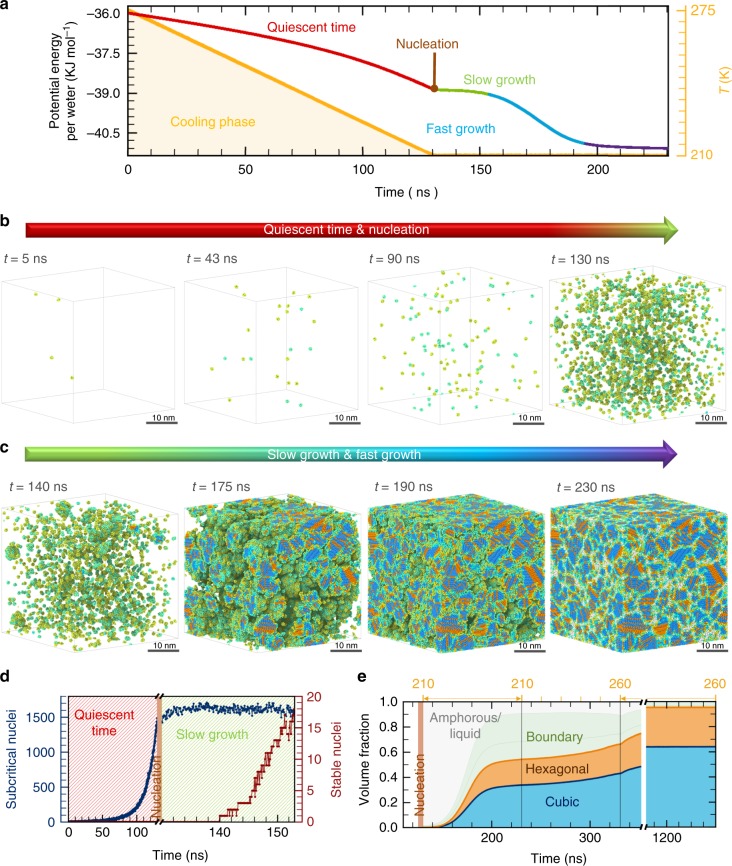
Homogeneous nucleation simulations of ice performed using ML-BOP_dih_. System dynamics and evolution of structural motifs during the cooling phase from homogeneous nucleation leading up to the grain boundary formation and grain growth (Supplementary Movie 1). **a** The total potential energy variation of the 2 million-water molecule system during the cooling phase from 275 to 210.5 K and at longer times when the system temperature is kept constant at 210.5 K. We identify four distinct stages: an initial quiescent time shown by the red line when no nucleation event occurs; the nucleation followed by an initial slow transformation shown by the slow energy decreasing period in green; a fast transformation phase of the grains shown by the rapid decrease in potential energy in blue; and a plateauing of potential energy shown in purple marks the completion of the phase transformation. **b** The snapshots show the subcritical water nuclei during the long quiescent phase leading up to the nucleation. The first nucleation event for the 2 million-water system occurs at *t* = 130 ns. Liquid water molecules are not shown for clarity. **c** MD simulation snapshots showing the various stages of grain growth and grain boundary during the post-nucleation stage. Blue, brown and green spheres represent cubic, hexagonal and amorphous ice, respectively. Liquid water is omitted for clarity. **d** The temporal evolution of the number of subcritical water nuclei (size <100 molecules) from the quiescent period and the initial appearance of stable nuclei during the post-nucleation stage. **e** The corresponding temporal evolution of the fraction of cubic and hexagonal ice

As a representative test case, we perform MD simulations on multi-million water molecules using ML-BOP models to understand at the molecular level homogeneous nucleation of supercooled water leading up to the formation and growth of grains of ice. Figure 4 summarizes the initial stages of nucleation leading up to the formation of polycrystalline ice for one such trajectory when water is slowly cooled from 275 to 210 K over 130.4 ns (cooling rate ~0.5 K ns^−1^). Following the appearance of the first stable nuclei at ~210 K, the temperature was held at 210 K for a further 100 ns to study the nucleation and growth processes in this homogeneously nucleated water. Figure 4a shows the potential energy variation as a function of time during the cooling phase and constant temperature phase. We identify four distinct stages during the freezing process: a long quiescent time period of ~130 nanoseconds before the first nucleation events; a period of slow transformation with a limited number of nuclei (13 at *t* = 150 ns, Fig. 4d); accelerated transformation driven by growth of a greater number of nuclei (~185 at 200 ns); and completion of grain growth to form a polycrystalline box of ice. Figure 4b shows the corresponding snapshots during the initial quiescent period when the system explores the relatively flat energy landscape before entering the nucleation and growth period. The molecular level illustration is consistent with classical nucleation theory; the quiescent period is marked by pronounced fluctuations of many subcritical nuclei which rapidly form, break and reform in the supercooled liquid as shown in Fig. 4d. The post-quiescent period shown by MD snapshots in Fig. 4c is marked by formation of multiple stable nuclei which grow slowly followed by a rapid growth phase when the grains begin to percolate through the entire three-dimensional space. The completion of the growth phase is characterized by the formation of a polycrystalline ice with the nanoscopic grains separated by boundaries comprised of amorphous ice. A local structure analysis (see Methods) of the growing structure reveals that the grains are comprised of stacking disordered ice (I_sd_) i.e., randomly mixed alternating sheets of hexagonal and cubic ice (see Supplementary Figure 7 for the local structure). Figure 4e shows that the evolving ice structure becomes increasingly rich in I_c_ phase compared to the more stable I_h_ phase with the ratio of cubic to hexagonal to be ~1.85 at the end of *t* = 350 ns. The observed preference for cubic ice formation is consistent with multiple experimental results in the past including a recent X-ray diffraction study^27^ and CG simulations^28^ as well as atomistic simulations using forward-flux sampling technique^29^.

### Nature of polycrystalline ice and transformation of stacking disordered ice to hexagonal ice

**Fig. 5:**
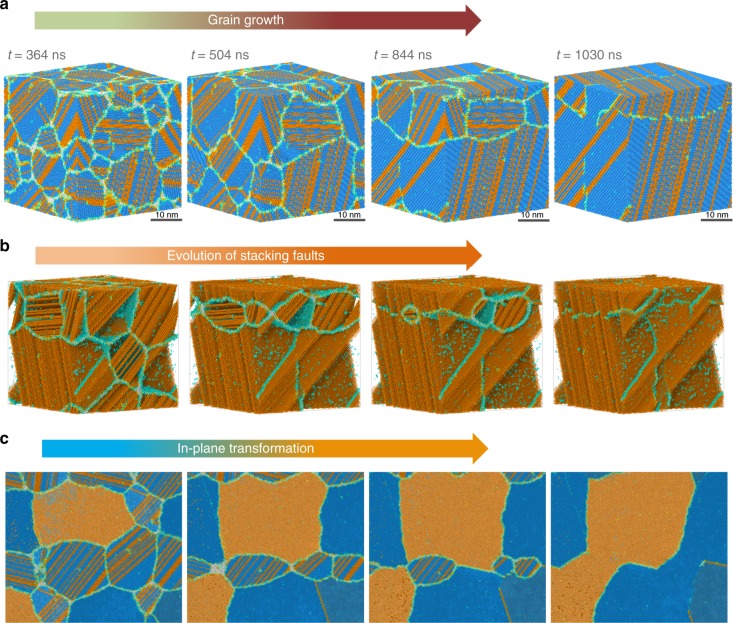
Post-nucleation ice grain growth simulations performed using ML-BOP_dih_. System dynamics and evolution of structural motifs of post-nucleation phase after the slow heating from 210 to 260 K. **a** Snapshots from simulations showing the grain growth process of nanosized grains at 260 K evolving into a single grain. (see Supplementary Movie 2 for a zoom-in view) **b** Snapshots from simulations (Supplementary Movie 3) showing the time evolution of hexagonal layers in stacking disordered ice. Cubic type molecules are not shown for clarity. Note that the ML-BOP model considers only nearest neighbor interactions but is able to reproduce the random stacking-disorder which is consistent with experimental observations^31^. **c** Snapshots from simulations (Supplementary Movie 4) showing the in-plane transformation between cubic and hexagonal layers in stacking disordered ice, viewed along the direction perpendicular to the basal plane of the largest stacking ice grain in the system

The final microstructure at 230 ns (Fig. 4c) is fine grained (average grain size ~9300 water molecules) and is expected to anneal over long times (microsecond to seconds) to naturally observed larger grains. We slowly anneal the nanocrystalline sample by heating from 210 to 260 K over 100 ns and then hold the sample at 260 K until the grains coarsen. The polycrystalline sample evolves into a single grain at the end of 1 microsecond of simulation (Fig. 5a). The internal structure of the grains is ice I_sd_, i.e., randomly mixed alternating sheets of hexagonal and cubic ice, comprised of stacking faults that evolve over time (Fig. 5b). The ice I_sd_ structure observed in our simulations is rich in I_c_ phase compared to the more stable I_h_ phase with the ratio of cubic to hexagonal being ~2 by 1200 ns (Fig. 4e).

The preference for ice I_sd_ formation during nucleation is consistent with recent experiments and simulations^27–30^ but the stacking disorder in polycrystalline ice has been much debated^27^. Kuhs et al.^31^ have analyzed neutron diffraction data and electron microscopy images to study the extent of stacking disorder in ice in the 170–190 K range. They tracked the evolution of cubicity as a function of time and note that the fraction of cubic stacking sequences is ~0.5. At temperatures > 180 K, cubicity decreases slowly to approach pure I_h_ after annealing over 10–12 h. Molinero and co-workers^28^ have analyzed the structure of ice that crystallizes at 180 K and shown that the ratio of cubic to hexagonal stacking sequences is ~2:1, which is similar to those found in our work. Indeed, more recent studies by Amaya et al.^32^ using femtosecond wide-angle x-ray scattering confirm that ice formed by nanodroplets that freeze rapidly at timescales of the order of 1 microsecond indeed have much higher cubicity values ~0.78 ± 0.05. This value is much higher than that reported by Kuhs et al.^31^. Nonetheless, these studies suggest that there can be a range of stacking disordered ice with different cubicity. The differences in the extent of stacking disorder were attributed to the differences in freezing temperatures^33^, the size of droplets (nanosized *vs*. micron sized) and the freezing rates^34^ (microseconds vs. seconds) to name a few.

Capturing the energetic ordering and the subtle energetic differences between ice phases within a molecular model remains a major challenge. While the stable phase at weak undercooling is I_h_, the ice phase that nucleates from supercooled water is, however, the stacking disordered ice. Free energy calculations performed by Molinero and co-workers^30^, using the mW model, show that the entropy of mixing of cubic and hexagonal layers makes stacking-disordered ice the stable phase for crystallites sizes up to 100,000 molecules. We note that the free energy cost of producing a growth fault in ice I_h_ for the mW model is ~15.3 ± 2.3 J mol^−1^ (0.159 ± 0.024 meV), which is consistent with the experimental value of 16.5 ± 1.7 J mol^−1^ (0.171 ± 0.018 meV) reported by Hondoh et al.^35^. Depending on the experimental conditions and the method of sample preparation, there is a range of free energy or enthalpy reported for the transformation of stacking disordered ice to pure hexagonal ice. For example, Ghormley et al.^36^ report transformation of cubic to hexagonal crystals to be ~22 J mol^−1^ (0.228 meV) in heating from 223 to 268 K. Differential scanning calorimetry of transformation of cubic ice (prepared by rapid quenching of liquid water at 190 K) to hexagonal ice report a slightly higher value ~56 J mol^−1^ (0.580 meV). On the other hand, McMillian et al.^37^ used calorimetry measurements and report a heat of transformation ΔH = 160 J mol^−1^ (1.658 meV) between ‘cubic’ and hexagonal ice. Likewise, Shilling et al.^38^ prepared amorphous ices by vapor deposition at 90 K and transformed them to stacking disordered ice by heating up to about 160 K. They report a free energy change of 155 ± 30 J mol^−1^ (1.606 ± 0.31 meV) for transforming I_sd_ to I_h_. This value is much higher than that reported by Ghormley et al.^36^. Differential thermal analysis also suggests heat release of similar order when I_c_ transforms to more thermodynamically stable I_h_^39^. Note that the mW predicted free energy is lower than the experimental values of Shilling et al.^38^ and McMillian et al.^37^. On the other hand, recent ab initio studies also suggest a thermodynamic preference for I_h_ compared to I_c_ (~1.4 meV per H_2_O arising from the difference in anharmonicity between cubic and hexagonal ice)^14^. While DFT-PBE may not be the best method for estimating the energetics of ice, this high free energy difference between I_c_ and I_h_ cannot be captured by nearest neighbor interactions as is the case in ML-BOP, ML-mW and mW. We, therefore, introduce an additional four-body term to the ML-BOP in the form of on-the-fly dihedrals model and retrained this ML-BOP_dih_ model by including the average energy difference between cubic and hexagonal ice (reported in ref. ^14^ using DFT-PBE) in the training data set. This 4-body term essentially captures the energetics difference provided by the PBE input data in ref. ^14^. One can retrain the 4-body term (Eqs. 8–10) if new improved ab initio data becomes available.

**Fig. 6:**
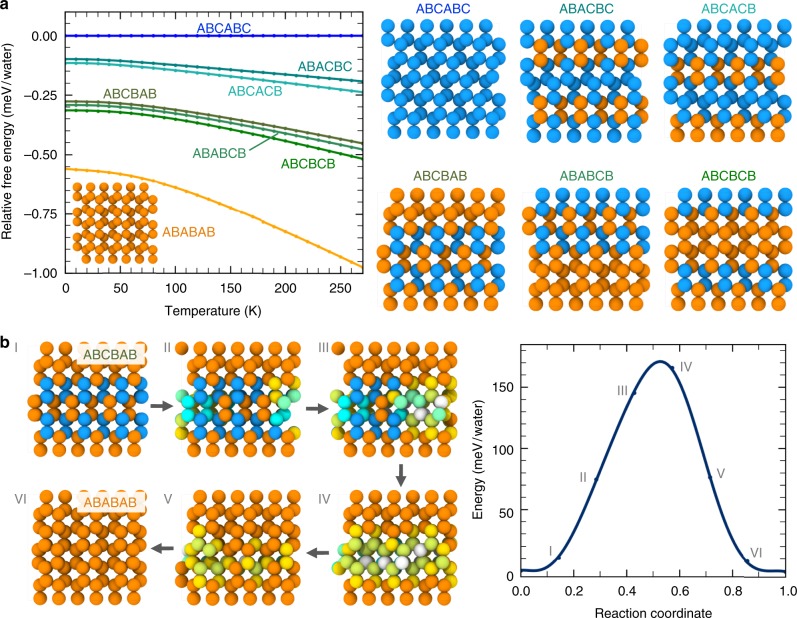
Thermodynamics of various ice phases and activation barrier computed using ML-BOP_dih_. **a** Free energies of various stacked disorder ice phases, I_c_, and I_h_ at different temperatures, relative to I_c_. Molecular configurations of the various phases are also provided. **b** Molecular pathway, and the associated energy barrier for elimination of a stacking fault plane in I_h_. Selected configurations along the pathway are shown on the left, while potential energy along this preferred pathway (obtained by CI-NEB calculations in the framework of ML-BOP_dih_) is shown on the right. All the molecules are colored based on their local coordination using the same scheme as Fig. 4

To test the thermodynamic preference of our new ML-BOP_dih_ model, we calculate the free energies of various ice phases (Fig. 6a) within the quasi-harmonic approximation (see Methods). Our model is able to capture the temperature-dependent stability of cubic, stacking disordered and hexagonal phases; hexagonal is the most stable phase and is ~1 meV per molecule lower than the metastable cubic phase at 260 K (Table 6). Despite I_h_ being energetically preferred compared to I_sd_, we do not observe a transformation to a pure hexagonal phase even after 1.3 μs of simulations possibly due to sufficiently large activation barrier. Indeed, climbing image Nudged Elastic Band (CI-NEB) calculations within the framework of ML-BOP_dih_ show that the energetic barrier associated with elimination of a stacking fault plane in hexagonal ice is ~170 meV (Fig. 6b). The atomic-scale pathway governing the transformation of a representative I_sd_ with ABCBAB stacking to I_h_ (ABABAB) is shown in Fig. 6b. The sliding of the molecules in the C-plane to their respective A-plane positions entails a range of concerted molecular motions involving stretching/rotation of hydrogen bonds. These coordinated movements result in localized strain in the vicinity of the stacking fault plane and underlie the activation barrier (~170 meV) associated with elimination of the fault. Thermal fluctuations at 260 K (kT ~ 22 meV) are sufficiently small to preclude observation of the I_sd_ → I_h_ transformation within μs timescales. In-plane I_sd_ → I_h_ transitions have much lower barriers (~5 meV per water) and are frequently observed at MD timescales (see Fig. 5c and Supplementary Movie 3, 4). Indeed, this behavior is consistent with the partial dislocation mechanism proposed by Hondoh et al.^40^. It is also worth noting that the hexagonal ice becomes thermodynamically more favorable as we approach the melting point; hence the free energy difference between I_h_ and I_c_ is expected to increase (as shown in Fig. 6a). The different stacking-disordered ice configurations have much smaller free energy difference (<0.2 meV per water), which can explain the presence of random stacking disorder observed in previous experiments^41^ and simulations^27,28,31,41–43^.

In summary, we introduced a machine learning strategy to train CG models, namely ML-BOP, ML-BOP_dih_ and ML-mW, for water simulations. As proof-of-principle, we use the developed ML CG models (ML-BOP and ML-BOP_dih_) to elucidate the mesoscale mechanism of ice grain formation and growth from supercooled water. In light of the accuracy and speed of our ML potential models, we foresee their wide usage in problems including phase transitions, homogeneous and heterogeneous nucleation, interfacial properties, co-existent regimes, and mechanical behavior, all at system sizes and times inaccessible to current popular models such as the TIP5P and TIP4P models.

## Methods

### Molecular dynamics simulations

Using the final ML-BOP and ML-BOP_dih_ potentials, we perform massively parallel, long-time MD simulations of super-cooled water to study the sequence of steps from nucleation to grain formation and coarsening. We start with a simulation cell containing 2,048,000 molecules of water at 275 K. Simulations with a larger sized cell containing ~8 million water molecules are also performed. In both the cases, the structure is minimized and equilibrated for 100 ps at 275 K in an NPT ensemble. Subsequently, the liquid water is cooled down to 200 K over 150 ns under isobaric conditions. We observe the first stable nuclei at ~210 K. Consequently, we stop the cooling at 210 K and hold the super-cooled liquid at 210 K for 100 ns. Following nucleation and growth of nanocrystalline ice grains at this temperature, we anneal the structure to 260 K at a rate of 0.5 K ns^−1^ and then hold it at 260 K beyond a microsecond to study the temporal evolution of grains.

### Identification of ice polytypes and grains

Hexagonal (I_h_), cubic (I_c_), and amorphous/liquid phases of ice are determined using a structure identification algorithm^44^ implemented in the visualization software OVITO^45^. The molecules are color coded according to their local environment (see Supplementary Figure 7). A feature detection algorithm based on image processing and unsupervised machine learning (clustering) techniques^46^ is developed to identify individual grains and their size distribution. The procedure involves voxelization (5 Å bin), contrasting filters, thresholding, DBSCAN clustering^47^, refinement, and position-based reverse mapping. All nearest neighbor searches are performed using a periodic k-d tree. This grain identification procedure accurately identifies small and large grains that are often irregularly shaped.

### Energy barrier calculations of stacking faults

The activation energy associated with the elimination of stacking fault plane in hexagonal ice is computed using Climbing Image Nudged Elastic Band (CI-NEB) calculations within the framework of ML-BOP_dih_ as implemented in LAMMPS^48–50^. For these calculations, the computational supercell consists of 6 layers (432 water molecules), with 72 molecules in the stacking fault plane.

### Free energy calculations

Free energies of the cubic, hexagonal, and stacking disorder ice polytypes are computed within the quasi-harmonic approximation^51^ using Phonopy^52^ to account for variation of phonon modes due to thermal expansion. For each ice phase, we first optimize the geometry of the unit cell in the framework of ML-BOP_dih_ until the energy difference between consecutive steps is <10^−4^ eV, and the atomic forces are within 10^−3^ eV Å^−1^. Next, we determine the changes in volume owing to thermal expansion using 1 ns long MD simulations at zero pressure and desired temperature. At each volume, phonon frequencies and related vibrational properties are computed via finite displacement approach using sufficiently large supercells (~ 88 Å × 70 Å × 66 Å) and displacement of 0.015 Å. The computed volume-dependence of phonon frequencies are then used to compute vibrational contribution to free energy at each temperature.

### Ice-liquid surface tension calculations

The liquid-ice surface tension is calculated using the mold integration method^53^. The well depth is chosen to be 10 meV based the value chosen for the original mW model^54^. The system consists of 1600 wells and a total of 6000 water molecules. The temperature for this calculation was chosen to be the freezing temperature for each of the respective models. The slab is 2 unit cells in thickness in the Z-direction which is the interfacial direction. To obtain the free energy vs well radius curve, a series of thermodynamic integrations are performed by incrementing the well radius by 0.1 Å increments. This covers a range from 0.4 Å up to 1.2 Å. These values were used to create a linear curve that could be used to extrapolate back to the optimal well radius to obtain the correct value for the surface tension. This is in line with the procedure outlined in ref. ^53^. After the curve is obtained, the optimal well depth for both systems is determined by running a simulation at each well radius and monitoring the crystallinity of the system. Each simulation is run for 10 ns. Once an approximate range is identified a set of intermediate radius values is examined to find the optimal well radius. We first benchmarked our procedure for the original mW and validate that the ice-liquid surface tension was 35 mJ/m^2^ consistent with that reported in refs. ^53,54^. For the ML-BOP and ML-BOP_dih_ it is found to be roughly 0.65 A while for the ML-MW it is found to be 0.45 Å, which yields the values in Table 4.

### Code availability

Code and workflow developed in this study are available from the authors upon reasonable request.

## Supplementary information

Supplementary Information

Description of Additional Supplementary Files

Supplementary Movie 1

Supplementary Movie 2

Supplementary Movie 3

Supplementary Movie 4

Supplementary Software

## Data Availability

The data that support the findings of this study are available from the authors upon reasonable request.
