# Supplementary material for: Machine learning coarse grained models for water
Source: Nat Commun. 2019 Jan 22;10:379. doi: 10.1038/s41467-018-08222-6 (PMC6342926; doi:10.1038/s41467-018-08222-6)
Supplement: Supplementary file 1 — Supplementary Information [file 41467_2018_8222_MOESM1_ESM.pdf]

Supplementary Information for
Machine learning coarse grained models for water Chan et al.

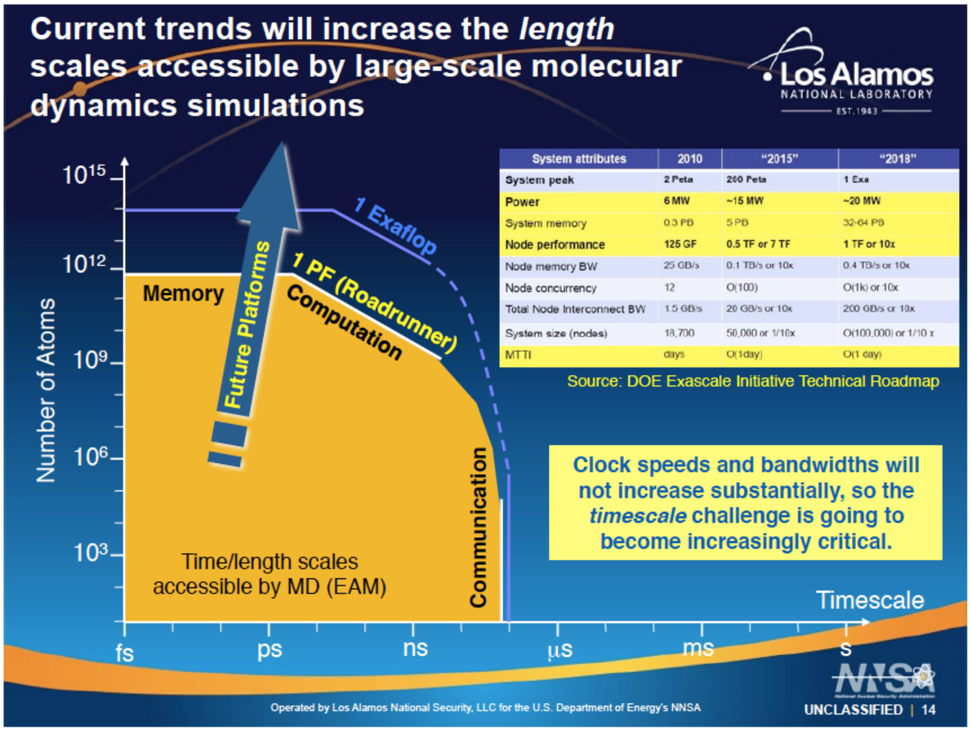

Supplementary Figure 1: Projections on the length scale and time scales accessible by large scale molecular dynamics simulations. (*Reproduced with permission from* Germann, Timothy C. *Exascale Co-design for Modeling Materials in Extreme Environments*. No. LA-UR-14-25062. Los Alamos National Laboratory (LANL), Los Alamos, NM (United States), 2014.**)**

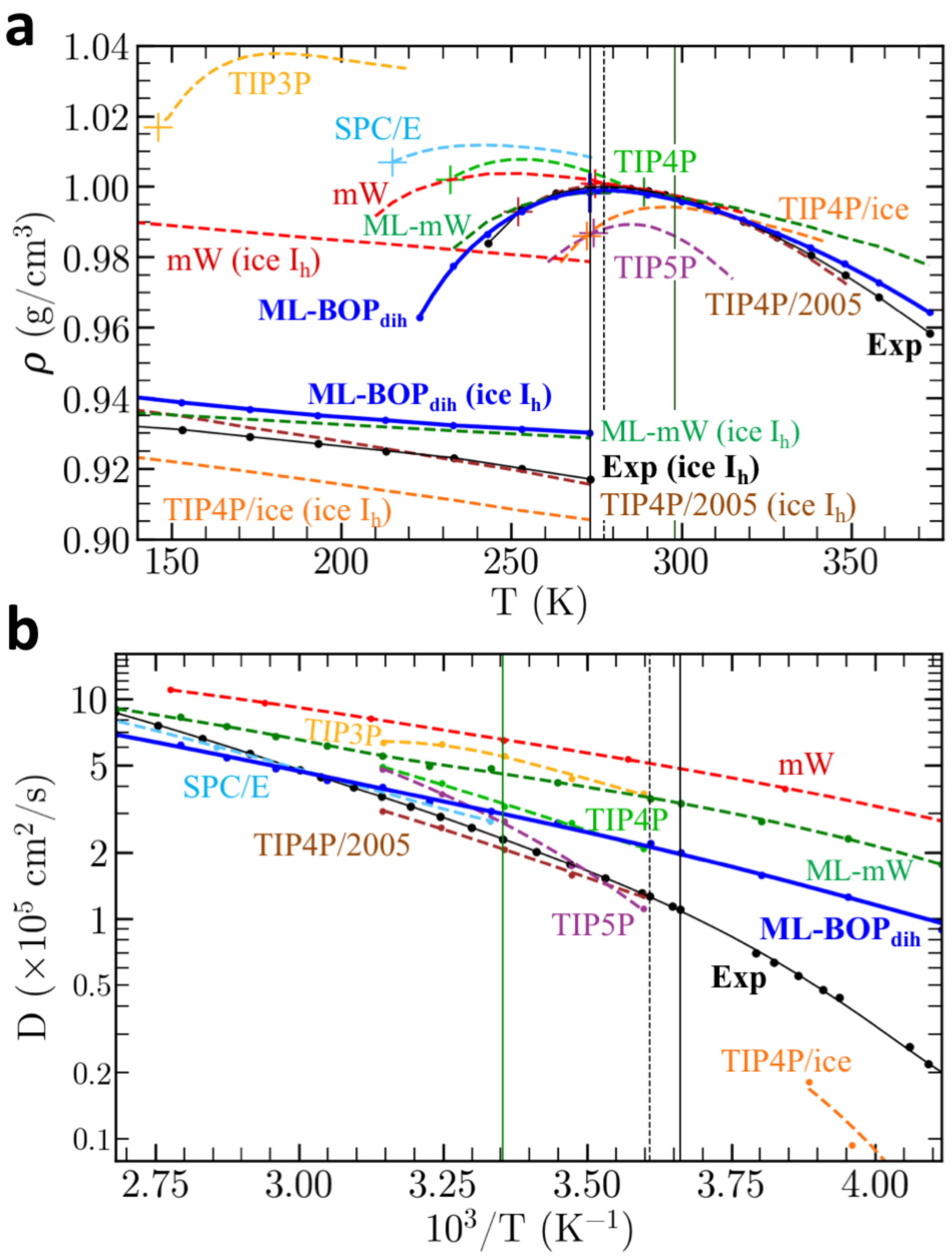

Supplementary Figure 2: Comparisons of the predictions by the ML-BOP models with those of other popular force fields. Predictive power is evaluated in terms of the ability to capture the density anomaly, diffusion coefficients, structural and other thermodynamic properties. Comparison data are extracted for mW[1], (TIP3P, TIP4P, TIP4P/2005, TIP5P)[2], and (SPC/E, TIP4P/ice)[3, 4]. The predictive power of ML-BOP models rival state-of-the-art atomistic model (TIP4P/2005) and outperforms most existing water models including coarse-grained models (mW). Melting point of water (T=273K), temperature of maximum density (T=277K), and room temperature (T=298K) are marked by a vertical solid black line, dotted black line, and solid green line, respectively. (**a**) The ML-BOP models correctly predict the melting point of water and the densities of ice and liquid water at pressure P = 1 bar over a wide range of temperature. A cross on the liquid density curve of each model marks their predicted melting points. (**b**) Diffusion coefficient of liquid water over a wide temperature range calculated using different models, and compared with experiments. The y-axis is shown in log scale.

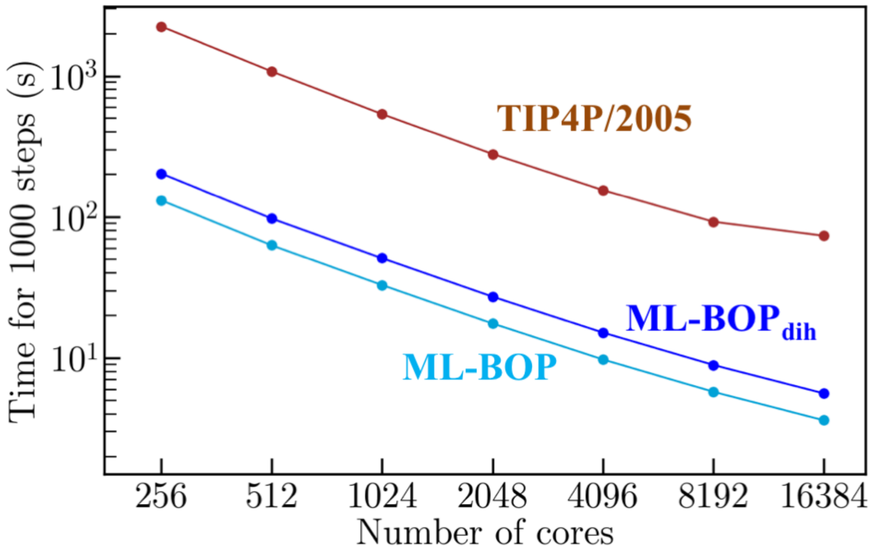

Supplementary Figure 3: Scaling data comparing the performance of the ML-BOP and ML-BOP$_{dih}$ model with that of TIP4P/2005 water model, which is the next most accurate potential model for water. The comparison is made for a system size of 256,000 water molecules and 1,000 MD time steps. The actual time step for comparable integration accuracy for the TIP4P/2005 potential is 5-10 times smaller than that of the ML-BOP$_{dih}$ model owing to the former's inclusion of high-frequency O-H vibrations. Therefore, to integrate to an equal amount of time, the ML-BOP$_{dih}$ model is ~ 120 times faster than TIP4P/2005 due to the factor of ~ 17 speed-up per time step (shown in the graph), and the additional factor of ~ 7 for larger time step that can be taken. ML-BOP is ~ 1.6 times faster than ML-BOP$_{dih}$.

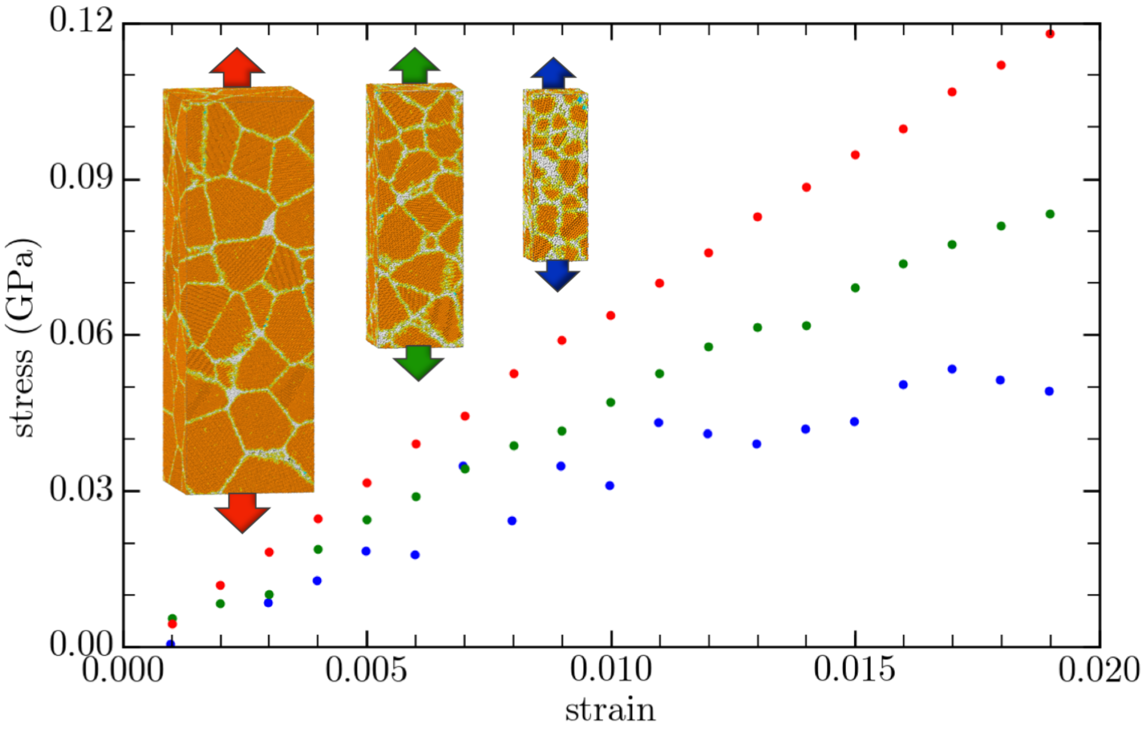

Supplementary Figure 4: Stress-strain curves (strain rate = 1×10^8 ps^{-1}) at 240 K for polycrystalline ice for samples with average grain sizes of 5 nm (blue circles), 10 nm (green circles) and 20 nm (red circles). We observe increased sample stiffness with increasing grain sizes, due to the small size of grains and consequently the large fraction of grain boundary atoms. This is consistent with studies of nanocrystalline copper with small grain sizes[5]. Inset shows snapshots of the samples showing the grain boundary mediated deformation.

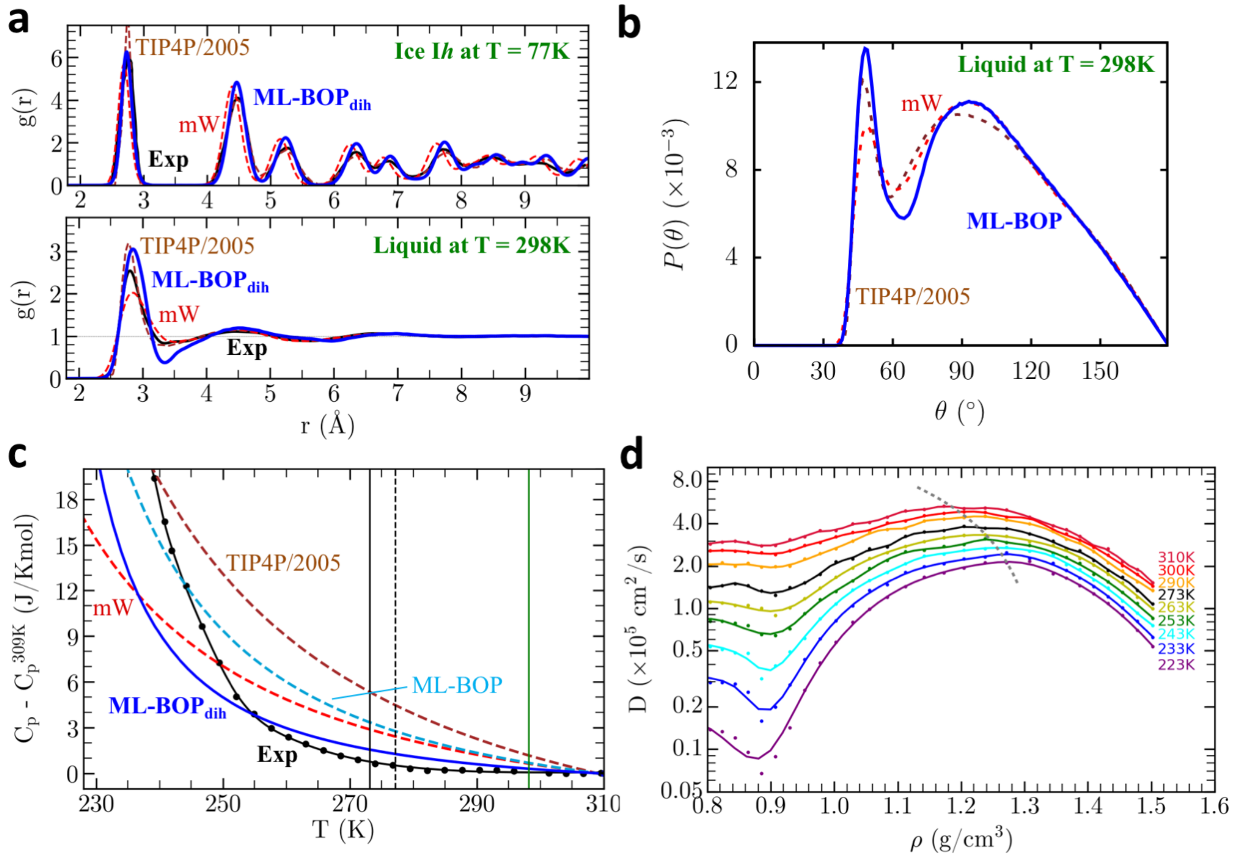

Supplementary Figure 5: Comparison of the structure and dynamical properties predicted by the ML-BOP models with those of other popular force fields. Comparison data are calculated or extracted for mW[1] and TIP4P/2005[2, 6]. Melting point of water (T=273 K), temperature of maximum density (T=277 K), and room temperature (T=298 K) are marked by vertical solid black line, dotted black line, and solid green line, respectively. (**a**) Comparison plot showing the radial distribution functions of ice at T=77 K and liquid water at T=298 K. (**b**) Comparison plot showing the angular distribution functions liquid water at T=298 K. (**c**) Comparison plot showing the relative heat capacity of water. The equation $f(a,b,c) = a\,(T/b - 1)^{-1.5} + c$ is used to extrapolate the data. All curves are offset by their values at T=309 K (Exp: 75.77, ML-ML-BOP$_{dih}$: 35.17, ML-BOP: 37.54, mW: 32.55, TIP4P/2005: 87.39 J K^{-1} mol^{-1}). (**d**) Plot showing the temperature-dependent, pressure-driven diffusion anomaly predicted by the ML-BOP models. The curves are cubic interpolation of the data points, which only serve as visual guides to show the overall trends. The dotted line shows the temperature dependent shift of the maximum.

Pressure dependence of g(r)

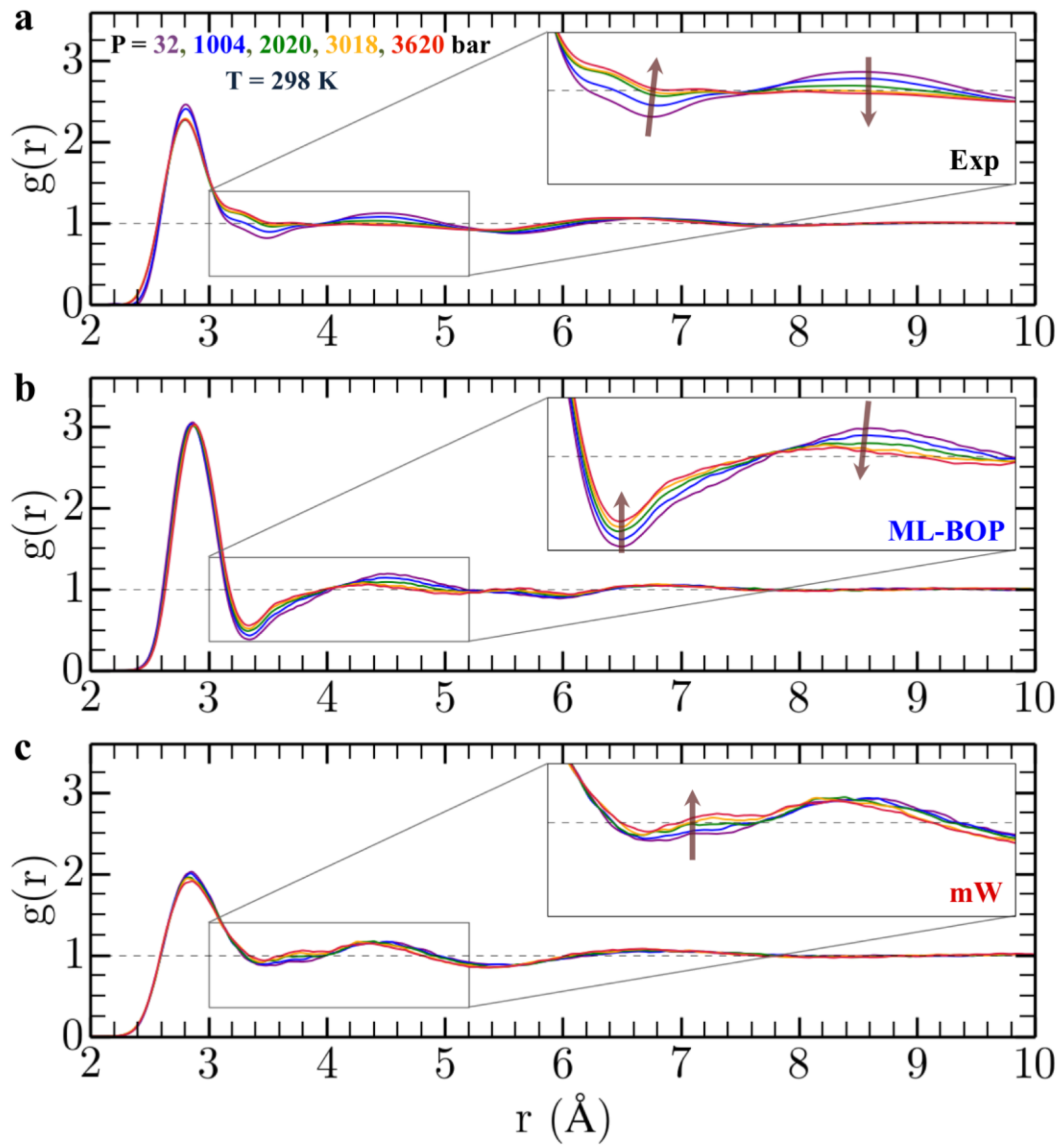

Supplementary Figure 6: Pressure dependence of O-O pair distribution functions at 298 K. Data for coarse-grained water models, ML-BOP (b) and mW (c), are calculated in the 32 to 3,620 bar pressure range and compared to experimentally measured data (a). The inset of each plot shows a magnified view of the 3 – 5.2 Å region and the arrows indicate the direction of increasing pressure.

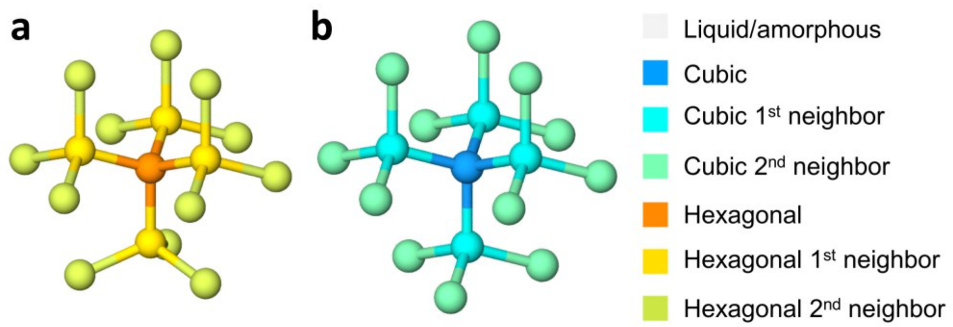

Supplementary Figure 7: Color coding to distinguish the local structure of the various molecules (a) Completely and partially hexagonal ice (b) Completely and partially cubic ice. Dark blue spheres represent completely cubic whereas turquoise and cyan represent partially cubic environments. Turquoise molecules represent partially cubic structures wherein the molecules are connected to at least one other cubic molecule in their first coordination. Cyan molecules represent partially cubic structures wherein the molecules are connected to at least one cubic molecule up to their second coordination. Orange spheres represent completely hexagonal while yellow and green represent partially hexagonal environments. Yellow molecules represent partially hexagonal structures wherein the molecules are connected to at least one other hexagonal molecule in their first coordination. Green molecules represent partially hexagonal structures wherein the molecules are connected to at least one hexagonal molecule up to their second coordination. White spheres represent amorphous or liquid like molecules.

Supplementary Table 1: Comparison between the parameters of ML-mW and mW models.

ML-mW

ϵ (eV)	σ (Å)	a	λ	γ	$\cos\theta_0$
0.297284	1.884015	2.124872	24.673877	1.207943	-0.279667

A	B	p	q	tol
7.111598	1.991526	4.011214	0.0	0.0

mW

ϵ (eV)	σ (Å)	a	λ	γ	$\cos\theta_0$
0.268376	2.3925	1.8	23.15	1.2	-0.333333

A	B	p	q	tol
7.04955628	0.602224558	4	0	0.0

Functional form of Stillinger-Weber:

$$E = \sum_i \sum_{j>i} \phi_2(r_{ij}) + \sum_i \sum_{j\neq i} \sum_{k>i} \phi_3(r_{ij}, r_{ik}, \theta_{ijk}) \quad (1)$$

$$\phi_2(\mathrm{r}_{ij}) = A_{ij}\epsilon_{ij}\left[B_{ij}\left(\frac{\sigma_{ij}}{r_{ij}}\right)^{p_{ij}} - \left(\frac{\sigma_{ij}}{r_{ij}}\right)^{q_{ij}}\right] e^{\left(\frac{\sigma_{ij}}{r_{ij}-a_{ij}\sigma_{ij}}\right)} \quad (2)$$

$$\phi_3(r_{ij}, r_{ik}, \theta_{ijk}) = \lambda_{ijk}\epsilon_{ijk}\left[\cos\theta_{ijk} - \cos\theta_{0ijk}\right]^2 e^{\left(\frac{\gamma_{ij}\sigma_{ij}}{r_{ij}-a_{ij}\sigma_{ij}}\right)} e^{\left(\frac{\gamma_{ik}\sigma_{ik}}{r_{ik}-a_{ik}\sigma_{ik}}\right)} \quad (3)$$

Supplementary Table 2: Hierarchy of the property classes used in HOGA optimization.

#	Hierarchy of property classes	Convergence Criteria
1	[a] Minimized structure of ice I_h, equation of state	RMSD < 0.7 Å , ΔE < 0.1 eV
2	[e] Time averaged density of Ice I_h at T= 273 K and P = 1 bar ($\rho_{Ih,273K,1bar}$)	$\lvert\rho_{predicted} - \rho_{exp}\rvert < 2\%$, SD < 0.01%
3	[e] Time averaged density of Ice I_h at T = 253 K and P = 1 bar ($\rho_{Ih,253K,1bar}$)	$\lvert\rho_{predicted} - \rho_{exp}\rvert < 2\%$, SD < 0.01%,
4	[e] Time averaged density of Ice I_h at T = 213 K and P = 1 bar ($\rho_{Ih,213K,1bar}$)	$\lvert\rho_{predicted} - \rho_{exp}\rvert < 2\%$, SD < 0.01%
5	[b] Relative ordering of $\rho_{Ih,1bar}$ at T = 213, 253, 273 K	$\rho_{Ih,273K,1bar} < \rho_{Ih,253K,1bar} < \rho_{Ih,213K,1bar}$
6	[e] Time averaged density of liquid at T= 338 K and P = 1 bar ($\rho_{liq,338K,1bar}$)	$\lvert\rho_{predicted} - \rho_{exp}\rvert < 2\%$, SD < 0.01%
7	[e] Time averaged density of liquid at T= 273 K and P = 1 bar ($\rho_{liq,273K,1bar}$)	$\lvert\rho_{predicted} - \rho_{exp}\rvert < 1.5\%$, SD < 0.01%
8	[d] Stability of ice I_h-liquid interface, melting point of ice I_h (T_m)	T_m within 20 K
9	[b] Enthalpy of melting of ice I_h (ΔH_{melt})	$\lvert\Delta H_{predicted} - \Delta H_{exp}\rvert < 30\%$
10	[e] Time averaged density of liquid at T= 300 K and P = 1 bar ($\rho_{liq,300K,1bar}$)	$\lvert\rho_{predicted} - \rho_{exp}\rvert < 1.5\%$, SD < 0.01%
11	[e] Time averaged density of liquid at T= 277 K and P = 1 bar ($\rho_{liq,277K,1bar}$)	$\lvert\rho_{predicted} - \rho_{exp}\rvert < 1\%$, SD < 0.01%
12	[e] Time averaged density of liquid at T= 263 K and P = 1 bar ($\rho_{liq,263K,1bar}$)	$\lvert\rho_{predicted} - \rho_{exp}\rvert < 3.5\%$, SD < 0.02%
13	[e] Time averaged density of liquid at T= 253 K and P = 1 bar ($\rho_{liq,253K,1bar}$)	$\lvert\rho_{predicted} - \rho_{exp}\rvert < 4\%$, SD < 0.02%
14	[b] Relative ordering of $\rho_{liq,1bar}$ at T = 253, 263, 273, 277, 300, 338 K	no more than 1 wrong order
15	[c] Enthalpy and free energy difference between ice I_h and I_c	(for ML-BOP_{dih} only)

[a] TIP4P/2005 data; [b] Experiment data; [c] ab initio data, Ref. 7; [d] indirectly fitted;
[e] TIP4P/2005 equilibrated starting configurations, experiment targets (see Table 6)

Supplementary Note 1: Pseudo code of the hierarchical objective function used in our machine learning workflow (HOGA).

```
# Define a list of properties to evaluate (with/without conditions)
properties = list (    structure of ice Ih at T=273K P=1bar (RMSD) to within 0.7 A,
                          density of ice Ih at T=273K P=1bar to within 0.02 g/cm3,
                          density of ice Ih at T=253K P=1bar to within 0.02 g/cm3,
                          …
                          correct relative ordering of ice densities at different T,
                          …
                          density of liq at T=300K P=1bar to within 0.05 g/cm3,
                          RDF of liq at T=300K P=1bar,
                          density of liq at T=277K P=1bar to within 0.005 g/cm3,
                          RDF of liq at T=277K P=1bar,
                          density of liq at T=273K P=1bar to within 0.01 g/cm3,
                          RDF of liq at T=273K P=1bar,
                          …
                          correct relative ordering of liq at different T,
                          density maximum at 277K,
                          melting point of water at P=1bar to within 3 K,
                          enthalpy of melting to within 0.3 kcal/mol,
                          diffusion coefficient of liq at T=373K P=1bar,
                          diffusion coefficient of liq at T=300K P=1bar,
                          …
                      )

# Set reward points and a maximum objective_value
reward = 1000   # arbitrary reward points for each property
max_objective_value = total_number_of_properties * reward

# Initialize objective value and counter
objective_value = 0
property_counter = 0

# Loop over the list of properties
for property in properties
{
run_necessary_MD_simulations()
sample_MD_trajectories()
calculate_averaged_property()
penality = calculate_score_based_of_fitness() # ideally less than reward
objective_value += penalty
if there is a condition and condition is not met
{
return max_objective_value - property_counter * reward + objective_value
terminate_objective_evaluation()
}
property_counter += 1
}

# Passed all evaluations! Return the final objective value.
return objective_value
```

Supplementary References

1. Molinero V, Moore EB. Water Modeled As an Intermediate Element between Carbon and Silicon. *J. Phys. Chem. B* **113**, 4008-4016 (2009).

2. Vega C, Abascal JLF, Conde MM, Aragones JL. What ice can teach us about water interactions: a critical comparison of the performance of different water models. *Faraday Discuss.* **141**, 251-276 (2009).

3. Vega C, Abascal JLF. Relation between the melting temperature and the temperature of maximum density for the most common models of water. *J. Chem. Phys.* **123**, 144504 (2005).

4. Lee SH. Temperature Dependence on Structure and Self-Diffusion of Water: A Molecular Dynamics Simulation Study using SPC/E Model. *Bull. Korean Chem. Soc.* **34**, 3800-3804 (2013).

5. Schiøtz J, Di Tolla FD, Jacobsen KW. Softening of nanocrystalline metals at very small grain sizes. *Nature* **391**, 561 (1998).

6. Pi HL*, et al.* Anomalies in water as obtained from computer simulations of the TIP4P/2005 model: density maxima, and density, isothermal compressibility and heat capacity minima. *Mol. Phys.* **107**, 365-374 (2009).

7. Engel EA, Monserrat B, Needs RJ. Anharmonic Nuclear Motion and the Relative Stability of Hexagonal and Cubic ice. *Phys. Rev. X* **5**, 021033 (2015).
