# Supplementary material for: Machine learning coarse grained models for water
Source: Nat Commun. 2019 Jan 22;10:379. doi: 10.1038/s41467-018-08222-6 (PMC6342926; doi:10.1038/s41467-018-08222-6)
Supplement: Supplementary file 2 — Description of Additional Supplementary Files [file 41467_2018_8222_MOESM2_ESM.pdf]

Description of Additional Supplementary Files

File name: Supplementary Movie 1
Description: Movie of a long-time MD trajectory showing the early stages of homogeneous ice nucleation and slow growth phase followed by a more rapid growth leading to the formation of nanocrystalline ice at 210 K.

File name: Supplementary Movie 2
Description: Movie of a MD trajectory showing a zoom-in view of the later stages of grain growth mechanism and consolidation *via* grain boundary migration.

File name: Supplementary Movie 3
Description: Movie of a MD simulation showing the dynamical transformation of hexagonal layers in stacking disordered ice during the grain growth process.

File name: Supplementary Movie 4
Description: Movie of a MD simulation showing an in-plane transformation at 260 K between cubic and hexagonal layers in stacking disordered ice.
